# Rodent‐mediated plant seed dispersal: What happens to the seeds after entering the gaps with different sizes?

**DOI:** 10.1002/ece3.8286

**Published:** 2022-01-11

**Authors:** Fei Yu, Guangjie Li, Shanshan Wei, Xianfeng Yi, Jianmin Ma, Keming Ma, Guangwen Chen

**Affiliations:** ^1^ College of Life Sciences Henan Normal University Xinxiang China; ^2^ State Key Laboratory of Urban and Regional Ecology Research Center for Eco‐Environmental Sciences Chinese Academy of Sciences Beijing China; ^3^ College of Life Sciences Qufu Normal University Qufu China

**Keywords:** gap size, rodent, scatter‐hoarding, seed dispersal, Taihang Mountains

## Abstract

In general, it is accepted that gap formation significantly affects the placement of scatter‐hoarded seeds by small rodents, but the effects of different forest gap sizes on the seed‐eating and scatter‐hoarding behaviors of small rodents remain unclear. Thus, we examined the effects of a closed‐canopy forest, forest edge, and gaps with different sizes on the spatial dispersal of *Quercus variabilis* acorns and cache placement by small rodents using coded plastic tags in the Taihang Mountains, China. The seeds were removed rapidly, and there were significant differences in the seed‐eating and caching strategies between the stand types. We found that *Q*. *variabilis* acorns were usually eaten after being removed from the closed‐canopy forest and forest edges. By contrast, the *Q*. *variabilis* acorns in the forest gap stands were more likely to be scatter‐hoarded. The dispersal distances of *Q*. *variabilis* acorns were significantly longer in the forest gap plots compared with the closed canopy and forest edge plots. However, the proportion of scatter‐hoarded seeds did not increase significantly as the gap size increased. In small‐scale oak reforestation projects or research, creating small gaps to promote rodent‐mediated seed dispersal may effectively accelerate forest recovery and successional processes.

## Graphical abstract

In general, it is accepted that gap formation significantly affects the placement of scatter‐hoarded seeds by small rodents, but the effects of different forest gap sizes on the seed‐eating and scatter‐hoarding behaviors of small rodents remain unclear. Thus, we examined the effects of a closed canopy forest, forest edge, and gaps with different sizes on the spatial dispersal of *Quercus variabilis* acorns and cache placement by small rodents using coded plastic tags in the Taihang Mountains, China. We found that the proportions of scatter‐hoarded seeds did not increase significantly as the gap size increased. In small‐scale oak reforestation projects or research, creating small gaps to promote rodent‐mediated seed dispersal may effectively accelerate forest recovery and successional processes.

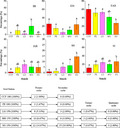

## 1 INTRODUCTION

Seedling establishment depends mainly on seed viability (Dassot & Collet, 2015), seed dispersal (Jansen et al., 2014; Yu et al., 2018), and microhabitat conditions which can determine the successful regeneration of tree species (Perea et al., 2011; Yu et al., 2013, 2018). In particular, seed dispersal is a key life‐history stage in plants, during which seeds or diaspores rely on agents such as wind and animals to spread to suitable sites away from the parent plant. Various studies have demonstrated that animals, especially small rodents, play a vital role in seed dispersal and they affect the seed‐to‐seedling period of plant regeneration (Abe et al., 2006; Williams‐Linera et al., 2011; Yu et al., 2017, 2018; Zhang et al., 2019, 2020). Usually, rodents eat part of the food (plant seeds or fruits) immediately after finding it, and the other part is often left scattered by the trees or stored to obtain food during periods of shortage (Ma et al., 2010). Not all buried seeds can be recovered by small rodents, and some seeds that escape predation may germinate and establish seedlings in suitable habitats (Abe et al., 2006; Li & Zhang, 2003; Lu & Zhang, 2004). Indeed, the scatter‐hoarding of seeds in suitable sites by rodents enhances the probability of seedling settlement (Vander Wall, 2001; Yu et al., 2015, 2017). Many factors may affect the quality and effectiveness of seed dispersal by animals, such as the microhabitat in the caching site (Wang & Corlett, 2017; Yang et al., 2016; Yu et al., 2014).

Gaps are frequent in various forests, and they have been shown to alter the microhabitat heterogeneity in forest ecosystems, thereby influencing the activity and foraging behavior of rodents, as well as seed germination and seedling establishment (Levey, 1988; Zhang et al., 2017). Many studies have investigated plant regeneration in forest gaps (Albanesi et al., 2008; Arevalo & Fernandez‐Palacios, 2007; Burnham & Lee, 2010; Yu et al., 2014; Zhu et al., 2014), but few have considered the interactions between seed predation and seed dispersal in forest gaps and the associated closed‐canopy forest.

Previous studies of the effects of gaps on rodent‐mediated seed dispersal obtained variable results. In some cases, small rodents can increase the likelihood of successful regeneration for gap‐dependent tree species by carrying the seeds of various tree species into forest gaps (Crawley, 1992; Iida, 2006). Previous studies have indicated that forest specialists avoid gaps (Bakker & Van Vuren, 2004; Rail et al., 1997; Rodríguez et al., 2001) because there is a higher perceived predation risk in open habitats (Lima & Dill, 1990). The predation risk is higher for animals in relatively open habitats (e.g., forest gaps) because they are easier to detect (Bélisle & Desrochers, 2002; Lima, 1998; Wilkinson et al., 2013). Wang and Corlett (2017) found that scatter hoarders prefer caching larger and more nutritious seeds in shrubs than in the open habitats, and the selection of caching microhabitat by small rodents was relevant to seed traits. However, some studies found that small rodents favor open habitats when selecting cache sites (Lichti et al., 2020; Steele et al., 2014, 2015) because less sheltered habitats reduce visits of other pilferers (Lichti et al., 2020; Steele et al., 2015). Canopy gaps are usually beneficial for seed dispersal. and they are more favorable sites for seed storage, thereby contributing to seedling establishment and survival (Hoshizaki et al., 1997; Iida, 2006). The size of forest gaps is important and it affects the maintenance of species diversity and forest regeneration (Wang et al., 2017). However, it is still not clear whether the sizes of gaps can affect the fate of seeds removed by rodents. The contradictory results obtained in previous studies may reflect variations in the stages of the co‐evolving plant–hoarder relationships.

Seed dispersal distance plays an important role in influencing seed fates and hoarding behaviors of small rodents (Liu et al., 2013; Perea et al., 2011; Wang & Corlett, 2017; Yu et al., 2018, 2020). It is generally considered that large seeds are dispersed further than small ones (Jansen et al., 2002; Yu et al., 2018, 2020), but compared with seed size, seed mass may have a greater effect on seed dispersal distance, and heavier seeds are dispersed further compared with light seeds (Jansen et al., 2004; Xiao et al., 2005a). However, the dispersal distance may be affected by not only seed traits (seed size, seed mass, seed coat thickness, nutritional value, and secondary chemicals) (Kuprewicz & García‐Robledo, 2019; Yi et al., 2015) but also the microhabitat (in both the origin and destination) (Perea et al., 2011; Wang & Corlett, 2017) and the number of dispersal movements (Perea et al., 2011). The gap size also has important effects on the seed dispersal of seeds produced by tree species and the success of germination (Van Ulft, 2004; Zhang et al., 2017). Recent studies have focused on the effects of forest gaps on forest regeneration, but the roles of forest gaps in seed dispersal by granivorous rodents are not fully understood, especially the relationship between the size of forest gaps and rodent‐mediated seed dispersal (Wang et al., 2017; Zhang et al., 2017). In particular, it is still unclear whether microhabitats such as gaps could increase the dispersal distances and seed survival.

In this study, we analyze the differences in the dispersal and predation of *Q*. *variabilis* seeds by scatter‐hoarding rodents in closed‐canopy forests, forest edges, and gaps with different sizes. We address the following two questions. (1) Are the decisions made by small rodents regarding scatter‐hoarding and the distribution of caches dependent on gap size? (2) Are large gaps preferred for rodent‐mediated seed dispersal? We hypothesize that the proportions of scatter‐hoarded seeds would not increase significantly with the gap size. Thus, we aim to obtain a better understanding of the effects of gap size on rodent‐mediated seed dispersal, thereby facilitating improved forest management.

## 2 MATERIALS AND METHODS

### 2.1 Study site

We conduct the experiment in the Huanglianshu Forest in the Taihang Mountains (112°25′E, 35°15′N), Jiyuan City, Henan Province, China. The study region is situated in the warm‐temperate zone where the annual precipitation ranges from 600 to 700 mm, most of which falls between July and September. Snow cover usually lasts five or more months (from November to March), and the mean annual temperature was 14.3°C. The forest was harvested during the 1960s and 1970s, and much of the area is now covered by secondary forests. The forest in this area has been protected against deforestation since the Taihang Macaque Natural Reserve was established in 1982. The secondary forest is dominated by *Q*. *variabilis* in the tree layer and by *Vitex negundo*, *Rosa xanthina*, *Rhamnus bungeana*, and *Cotinus coggygria* in the understory vegetation. *Apodemus peninsulae*, *Niviventer confucianus*, and Père David's rock squirrel (*Sciurotamias davidianus*) are common seed predators in the study region.

### 2.2 Abundance and species composition of small rodents

At the experimental site, we used 50 steel‐wire live traps (30 cm × 25 cm × 20 cm) baited with peanuts to capture and identify the rodent species that potentially removed the released seeds. Traps were placed along each of two transects at 5‐m intervals on September 23–26, 2017 (after the seed release experiment). Trap inspections were performed twice each day at sunrise and sunset. The captured animals were weighed and released. The total trapping effort = 50 traps × 3 days = 150.

### 2.3 Seed marking

We collected mature and fresh acorns of *Q*. *variabilis* seeds (acorns) from the ground outside our experimental stands for field release during 2016. Water flotation and visual inspection were employed to distinguish sound from insect‐damaged or empty acorns. In total, 810 *Q*. *variabilis* acorns (1.97 × 1.68 cm, 3.58 ± 0.21 g, *n* = 50) were randomly selected and labeled according to the plastic‐tagging methods reported by Zhang and Wang (2001) and Li and Zhang (2003) with slight modifications. A hole with a diameter of 0.3 mm was drilled through the husk near the germinal disk of each seed, but without damaging the cotyledon and embryo. Flexible plastic tags (3.0 × 1.0 cm, <0.1 g) were tied to the seeds by passing thin steel thread with a length of 10 cm through the hole. Each seed was marked with a unique numbered tag to ensure that seeds could be readily relocated and identified. The tags were frequently still visible on the surface of the ground after their burial in the soil or leaf litter by rodents, which made them easy to find. It has been shown that the effects of tagging on the seed removal and hoarding behaviors of rodents are negligible (Kempter et al., 2018; Xiao et al., 2006).

### 2.4 Seed release and seed removal

To examine the effects of different forest stand types, that is, closed‐canopy forest (CCF), forest edge (FE), and gaps with different sizes, on the spatial dispersal of *Q*. *variabilis* acorns and cache placement by small rodents, 10 approximately elliptical gaps with various sizes were selected 300 m apart in the different mountain slopes in secondary pure *Q*. *variabilis* forests at the end of 2015 (winter) with an area of about 10.0 ha, that is, two large gaps measuring >500 m^2^ (LG), three medium gaps measuring 500–150 m^2^ (MG), and five small gaps measuring <150 m^2^ (SG), and these forest stand types have similar site conditions, with a similar soil type (typical brown forest), topography, history of forest management, interference conditions, and vegetation conditions. Seed dispersal experiments began in 2016.

**FIGURE 1:**
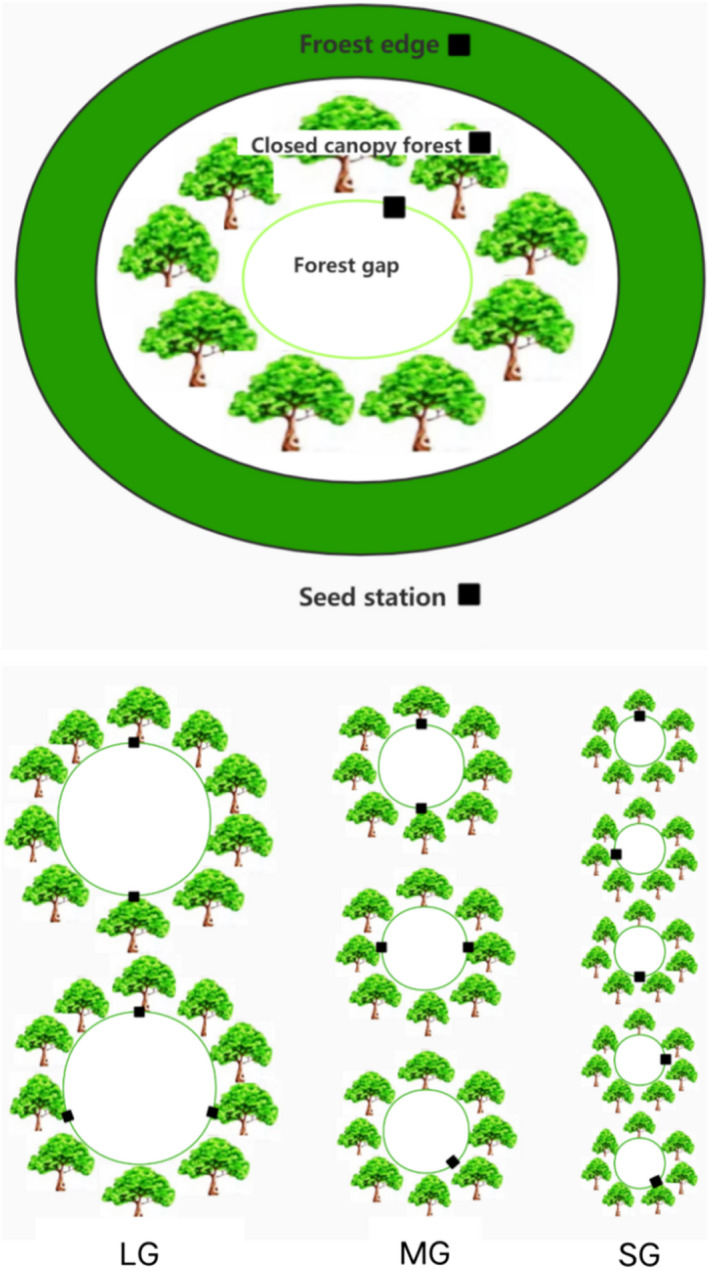
Map showing the locations of the seed stations in the experimental plots

In CCF and FE, a transect was set along with the mountain trend respectively, six seed stations (1 m × 1 m) were distributed along the transect lines 50m apart from each other, and we set 5 seed stations in LG, MG, and SG. The tagged acorns were evenly placed throughout each station. That is, we set six seed stations in CCF and FE, and set five seed stations in LG, MG, and SG. In total, 27 seed stations were set 50 m apart at the study site (Figure 1). We placed 30 tagged seeds at each separate seed station. The total number of seeds released was 27 (stations) × 30 (seeds) = 810 seeds. From the day after seeds were released, we checked each station daily for seed removal until all seeds were removed or consumed. We ensured that the search time of each seed station is not <30 min and tried to search the area around each seed station in order to record the fate of each tagged seeds. During each visit, we inspected each seed station as well as the caches found in previous visits. The postdispersal seed fates were classified using six categories (Yi & Zhang, 2008): (1) intact in situ (IIS); (2) eaten in situ (EIS); (3) moved and eaten leaving only plastic tags and seed fragments (EAR); (4) intact but not buried after removal (IAR); (5) scatter‐hoarding after removal (SH); and (6) missing where their true fates were unknown (M). When a cache was found, we recorded the seed tag numbers and measured the distances of the tagged seeds from their original seed stations, and a chopstick was coded with the same number as the tag and placed 25 cm away from the seed cache sites to mark each cache location. During the next visit, we also surveyed the caches located in previous visits until the caches were removed or eaten by rodents. The areas around the caches were determined by selecting a random angle from the caches and a random distance within the 50‐m radius search area around the seed caches when marked caches were recached.

### 2.5 Data analysis

SPSS for Windows (version 18.0) was used to conduct the statistical analyses. We compared the numbers of remaining, eaten, and cached seeds, where each was divided by the total number of seeds released. To achieve normality and homogeneity of variance, the proportions of remaining, eaten, and cached seeds were transformed using the arcsine‐square‐root transformation for the following data analysis. Cox regression analysis was used to test for differences in the seed removal rates among the five types. A univariate generalized linear model was employed to identify the effects of stand types on the seed dispersal distance and the six seed fates. Closed‐canopy forest (CCF), forest edge (FE), and gap size (large, medium, and small gaps) were used for fixed effects, random effects in the models included seed stations position, risks of predation, and pilferage by other animals (Perea et al., 2012; Steele et al., 2015; Yu et al., 2015; Zhang et al., 2008). Tukey's honest significant difference post hoc tests were performed for multiple comparisons.

## 3 RESULTS

### 3.1 Identification of seed‐removing rodents

We captured 31 rodents and *Apodemus peninsulae*, *Sciurotamias davidianus*, and *Niviventer confucianus* accounted for 64.5%, 9.7%, and 25.8% of all the captures, respectively. We did not capture any birds in this study, so we have no data on Eurasian jays at our study site. However, the Eurasian jay (*Garrulus glandarius* Linnaeus) was previously observed and it is considered as a probable species that disperses and forages acorns and pine seeds.

### 3.2 Removal rates from seed stations

**FIGURE 2:**
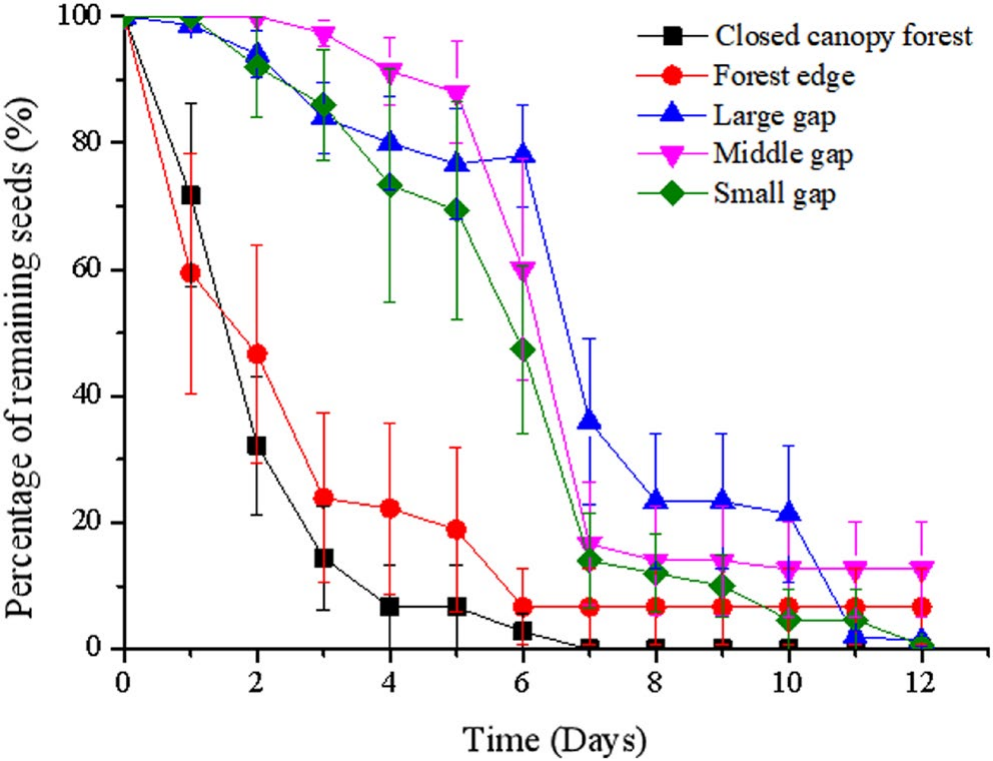
*Quercus variabilis* seed removal rates from the seed stations in the five stand types. Data represented as mean ± standard error

Most of the acorns released in CCF and FE were eaten or removed by small rodents within six days after release (Figure 2). By contrast, only 22.0%, 40.0%, and 52.7% of the seeds released in the LG, MG, and SG stands, respectively, were eaten or removed by small rodents. The stand type had a significant effect on the removal rate for the seeds handled by animals (Wald = 36.142, *df* = 4, *p* < .001).

Cox regression analysis shows that the seed removal speed was significantly higher in CCF than those in SG (Wald = 21.491, *df* = 1, *p* < .001), MG (Wald = 32.601, *df* = 1, *p* < .001), LG (Wald = 6.483, *df* = 1, *p* = .011), and FE (Wald = 5.499, *df* = 1, *p* = .019), respectively (Figure 2).

Cox regression analysis indicated that the seed removal speed in FE was significantly higher than those in SG (Wald = 8.364, *df* = 1, *p* = .004) and MG (Wald = 9.672, *df* = 1, *p* = .008) but not different to that in LG (Wald = 3.362, *df* = 1, *p* = .067) (Figure 2). However, Cox regression detected no significant differences in the seed removal speed between the stands with different gap sizes (Wald = 4.263, *df* = 2, *p* = .119).

### 3.3 Seed fates

**FIGURE 3:**
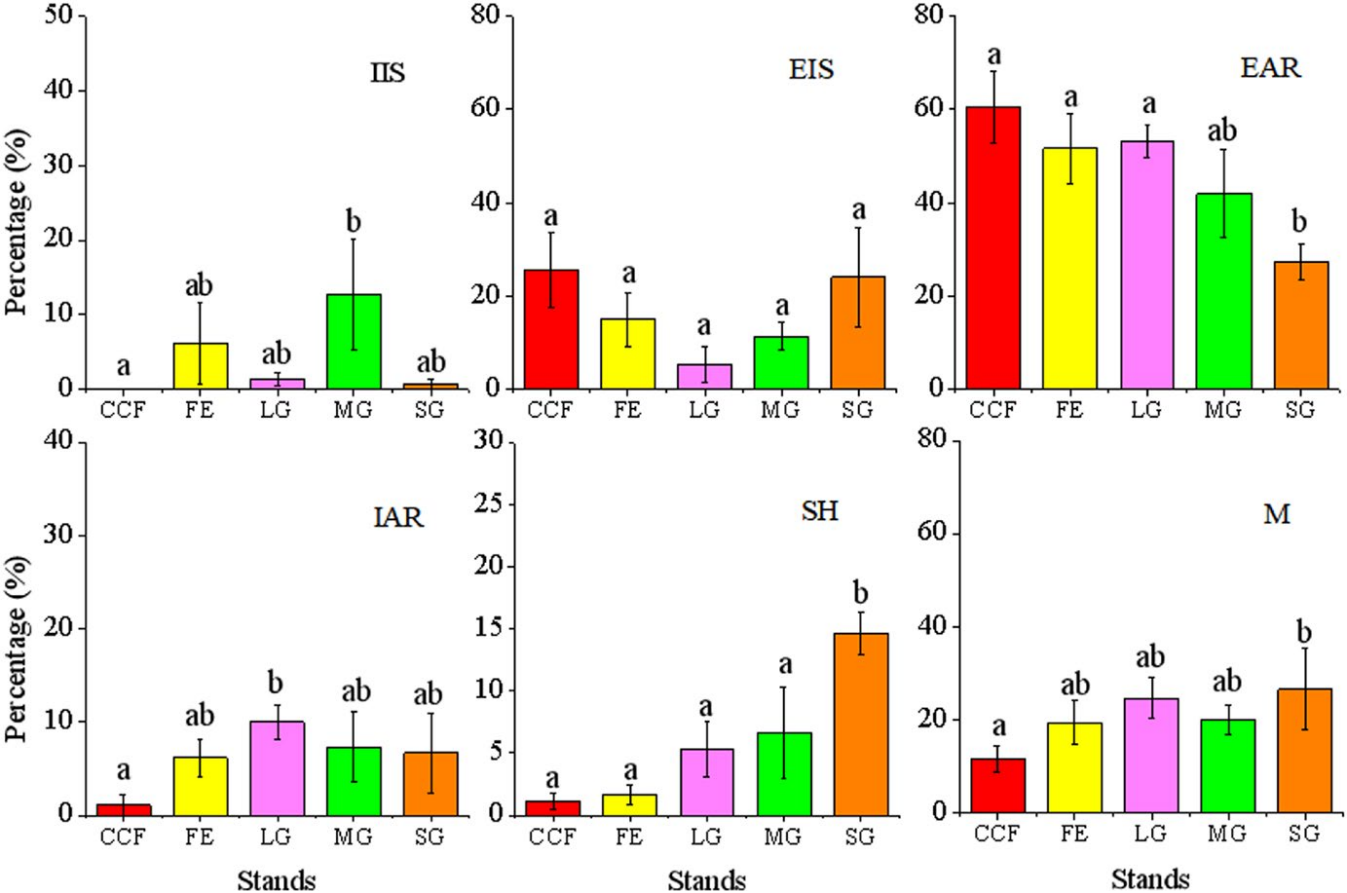
Fates of *Quercus variabilis* seeds after dispersal by small rodents in the five stand types. Data represented as mean ± standard error. Note: Different letters represent the significant differences in different treatments, with a significant level of *p* < .05, the same below

Significant differences were found in the proportions of EAR and SH among the five stands (EAR: *F* = 3.239, *df* = 4, *p* = .031; SH: *F* = 7.555, *df* = 4, *p* = .001), but there were no significant differences in the proportions of IIS, EIS, IAR, and M (IIS: *F* = 1.576, *df* = 4, *p* = .216; EIS: *F* = 1.504, *df* = 4, *p* = .235; IAR: *F* = 1.460, *df* = 4, *p* = .248; M: *F* = 1.378, *df* = 4, *p* = .274) (Figure 3).

**FIGURE 4:**
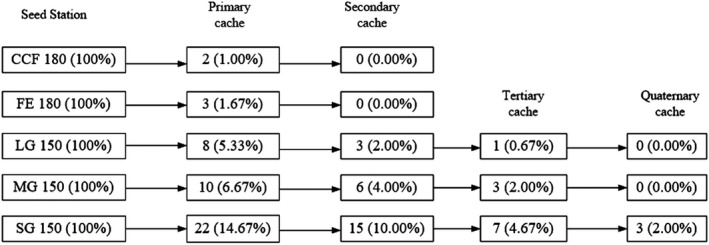
Scatter‐hoarding pathways for 810 tagged *Quercus variabilis* seeds from the seed stations in the five stand types

A higher proportion of seeds was SH in SG compared with CCF, FE, MG, and LG (SG vs CCF, *p* < .001; SG vs. FE, *p* < .001; SG vs. LG, *p* = .004; SG vs. MG, *p* = .011) (Figures 3 and 4) By contrast, slightly higher proportions of the seeds were SH in LG and MG than FE and CCF, but the differences were not significant (LG vs. FE, *p* = .142; LG vs. CCF, *p* = .200; MG vs. FE, *p* = .057; MG vs. CCF, *p* = .084) (Figure 3).

A lower proportion of seeds was EAR in SG compared with CCF (SG vs. CCF, *p* = .003), FE (SG vs. FE, *p* = .027), and LG (SG vs. LG, *p* = .026). A slightly lower proportion of the seeds was EAR in SG than MG, but the difference was not significant (SG vs. MG, *p* = .181) (Figure 3). Among the 810 seeds released, only one seed survived from an SG site and it was cached 4 times during the following spring.

### 3.4 Seed dispersal distance

**FIGURE 5:**
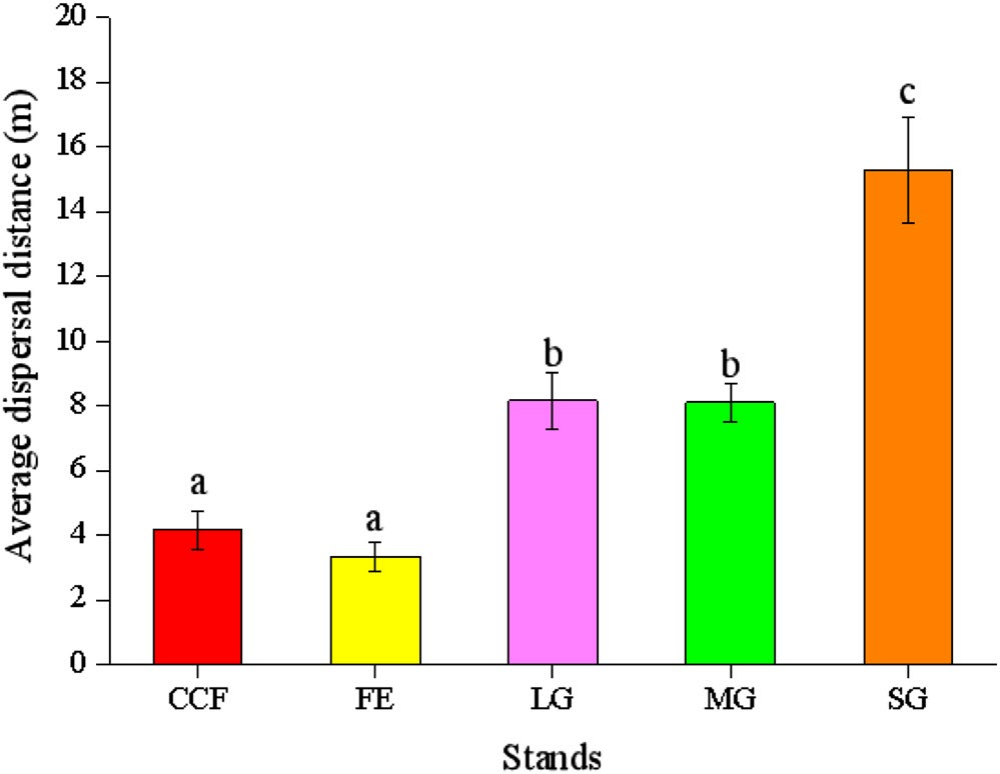
Dispersal distances of *Quercus variabilis* seeds after primary dispersal from the seed release stations in the five stand types. Data represented as mean ± standard error

Most of the seeds were dispersed within a distance of 10 m (Figure 5). The average dispersal distance was significantly affected by the stand types (*F* = 22.444, *df* = 4, *p* < .001) (Figure 5). The dispersal distances were significantly greater in SG, MG, and LG than CCF (SG vs. CCF, *p* < .001; MG vs. CCF, *p* = .027; LG vs. CCF, *p* = .031) and FE (SG vs. FE, *p* < .001; MG vs. FE, *p* = .008; LG vs. FE, *p* = .010). The dispersal distances were significantly greater in SG than MG (*p* < .001) and LG (*p* < .001). By contrast, there was no significant difference in the dispersal distances between MG and LG (*p* = .979).

## 4 DISCUSSION

Our results show that the seed removal speed was significantly higher in CCF than the other four stand types. These differences may have been due to the simpler vegetation structure in the gaps leading to decreased seed dispersal services from scatter‐hoarding rodents compared with CCF. In addition, the seed production rate was higher within the closed canopy than the gaps due to the lack of advanced regeneration, which also led to a higher speed of seed removal in CCF. Most of the released acorns were harvested rapidly after their placement by small rodents in the CCF and FE stands, thereby demonstrating small rodents are important for the effective dispersal of this type of seed. We found that the seeds from seed stations were carried rapidly by rodents, as shown in previous studies (Caccia et al., 2006; Chang et al., 2012; Jansen & Forget, 2001; Vander Wall, 1990; Xiao et al., 2005b; Yu et al., 2018, 2020). There were no significant differences in the seed removal rates among the stands with different gap sizes. Our results differ from those obtained in other studies where the gap size had a positive effect on the seed removal rate (Van Ulft, 2004; Wang et al., 2017). This difference may be explained by the similar shrub coverage and plant resources in the gaps.

In the present study, we found that compared with CCF and FE, more seeds were cached and less seeds were eaten after being removed from the LG, as also found in previous studies (Wang et al., 2017; Yang et al., 2016). The results clearly support the hypothesis tested in this study because the proportion of scatter‐hoarded seeds did not increase significantly as the gap size increased, possibly because rodents must trade‐off the risks of predation and pilferage by other animals (Lichti et al., 2020; Steele et al., 2015; Yang et al., 2016). A gap environment with large canopy openness increases the danger coefficient for the main seed disperser; they would rather scatter hoard to protect the seeds from pilfering rather than eat the seeds in dangerous gaps. In addition, the open environment in gaps provides a convenient condition for rodents to retrieve the cached seeds. This is because cached seeds with exposed labels are more detectable in the gaps (Steele et al., 2015; Yang et al., 2016; Yu et al., 2014). The seed fates might have varied between shrubs and open habitats because of differences in the activities and foraging behavior of rodents (Den Ouden et al., 2005; Perea et al., 2011; Perez‐Ramos & Maranon, 2008). The foraging behavior of mammals is associated with assessments of foraging costs and benefits, including time, energy, and the predation risk (Lichti et al., 2020; Schmidt & Ostfeld, 2003; Steele et al., 2015). The time spent on in situ eaten of seeds is usually longer than the time spent on removal, and the longer a seed disperser stays in the open, the higher the risk of predation. (Yu et al., 2014). The low ground coverage in large gaps may have affected the encounter rates with seeds, thereby affecting the seed predation and hoarding behaviors of rodents by increasing the predation risk estimated by rodents (Cintra, 1997). In addition, our previous research and a study by Wang et al. (2017) concluded that the proportion of seeds cached in canopy gaps was significantly lower than that in the understory (Yu et al., 2014). No seeds were actually provided in the gaps so the results may have been influenced by the fact that the seeds were offered only in the understory habitat. However, both of these studies found that the survival rates were higher in the gaps than CCF because of the more suitable environmental conditions (especially sufficient light) and lower risk of pilferage.

We found that the dispersal distances were significantly greater in the gaps than CCF and FE, and a previous study obtained similar results. It was also shown that an open microhabitat has positive effects on the dispersal distance and seed survival (Perea et al., 2011). In addition, Steele et al. (2014) found that squirrels tend to hide larger acorns further from the tree crowns, that is, in open habitats. Previous quantitative studies demonstrated that heavier acorns were dispersed further compared with light acorns (Jansen et al., 2004; Xiao et al., 2005a), and this does not support the energy‐saving hypothesis because of the effect of seed traits, for example, seed coat thickness, nutritional value, and rodent species on dispersal distance (Yi et al., 2015). In fact, both the origin and destination microhabitats, that is, abiotic factors, were more suitable for confirming dispersal distances.

All of the primary caches were recovered in CCF, FE, and SG and subsequently predated by rodents. Two seeds in the primary caches were cached in both MG and LG according to the last survey. Only one seed survived until the seedling stage and it emerged in SG during the following spring. Our observations agree with previous studies where only 0.02%–10% of the removed seeds established seedlings (Hulme, 2002; Jansen et al., 2002), and because flagged acorns are removed and eaten by rodents, with very few surviving to germination. Thus, the foraging behavior and visitation frequencies of rodents may have been higher in SG compared with LG. Our results demonstrate that small gaps had a positive effect on the hoarding of *Q*. *variabilis* seeds. However, the effect of the gap size on seed dispersal may vary according to the plant species and this requires further study.

Seed removal remains high and relatively constant over time, but partial seed damage (nonlethal) by rodents, as well as their caching and scatter‐hoarding behavior, and the satiation effect could result in more seeds transitioning to the seedling stage (Martínez‐Ramos et al., 2016). Our observations demonstrate that it is more important to consider both the origin and destination habitats when determining the seed dispersal distances and survival. Due to the low number of scatter‐hoarded seeds, increasing openness will reduce the probability of seed survival, thereby resulting in a higher probability of either partially or totally eaten seeds.

## 5 CONCLUSIONS

Our results clearly demonstrate that the gap size is an important factor that determines whether seeds are removed rapidly by predators or potential dispersers. Variations in the gap size can lead to different seed fates, which may eventually influence tree regeneration. We found that *Q*. *variabilis* acorns were usually eaten after their removal in CCF and FE. By contrast, the *Q*. *variabilis* acorns were more likely to be scatter‐hoarded in forest gap stands. Moreover, the proportion of scatter‐hoarded seeds did not increase significantly as the gap size increased. The *Q*. *variabilis* acorns in forest gap stands were dispersed significantly greater distances compared with those in CCF and FE. These results show that forest gaps can influence scatter‐hoarding decisions and the distribution of caches by small rodents. Thus, in small‐scale *Q*. *variabilis* reforestation projects or research, creating some small gaps to promote rodent‐mediated seed dispersal may be an effective method for accelerating forest recovery and successional processes. However, in the present study, only five small, three medium, and two large sizes of larch gaps were established, because of restrictions by national forestry policies and regulations in China (Lu et al., 2015). Therefore, the conclusions of the effects of gap size on animal‐mediated seed dispersal are limited. Although most studies have studied the immediate caching and dispersal fate of seeds using plastic tagging methods, they are generally not effective in assessing seed and seedling storage recovery and patterns, in addition to the effect of the habitat type might be complex so the general applicability of our findings requires further study. There must be a long‐term (e.g., more than five years) monitoring of seed dispersal to identify the influence of seed station position, and we plan to extend our study to explore the possible trade‐offs between dispersal capacities and other important ecological factors over diverse scales in terms of space and time.

## CONFLICT OF INTEREST

The author declares that he has no competing interests.

## AUTHOR CONTRIBUTIONS

**Fei Yu:** Conceptualization (lead); Data curation (lead); Funding acquisition (lead); Investigation (lead); Writing‐original draft (lead). **Guangjie Li:** Methodology (equal); Writing‐original draft (equal); Writing‐review & editing (equal). **Shanshan Wei:** Data curation (equal); Investigation (equal); Writing‐original draft (equal). **Xianfeng Yi:** Conceptualization (lead). **Jianmin Ma:** Investigation (equal); Writing‐original draft (equal). **Keming Ma:** Writing‐review & editing (equal). **Guangwen Chen:** Writing‐review & editing (equal).

## Data Availability

The data associated with this manuscript are available from the Dryad Repository (https://doi.org/10.5061/dryad.g1jwstqpm).
